# Bleeding Risk With Combination Intrapleural Fibrinolytic and Enzyme Therapy in Pleural Infection: An International, Multicenter, Retrospective Cohort Study

**DOI:** 10.1016/j.chest.2022.06.008

**Published:** 2022-06-16

**Authors:** Jason Akulian, Eihab O. Bedawi, Hawazin Abbas, Christine Argento, David T. Arnold, Akshu Balwan, Hitesh Batra, Juan Pablo Uribe Becerra, Adam Belanger, Kristin Berger, Allen Cole Burks, Jiwoon Chang, Ara A. Chrissian, David M. DiBardino, Xavier Fonseca Fuentes, Yaron B. Gesthalter, Christopher R. Gilbert, Kristen Glisinski, Mark Godfrey, Jed A. Gorden, Horiana Grosu, Mridul Gupta, Fayez Kheir, Kevin C. Ma, Adnan Majid, Fabien Maldonado, Nick A. Maskell, Hiren Mehta, Joshua Mercer, John Mullon, Darlene Nelson, Elaine Nguyen, Edward M. Pickering, Jonathan Puchalski, Chakravarthy Reddy, Alberto E. Revelo, Lance Roller, Ashutosh Sachdeva, Trinidad Sanchez, Priya Sathyanarayan, Roy Semaan, Michal Senitko, Samira Shojaee, Ryan Story, Jeffrey Thiboutot, Momen Wahidi, Candice L. Wilshire, Diana Yu, Aline Zouk, Najib M. Rahman, Lonny Yarmus, Interventional Pulmonary Outcomes Group

**Affiliations:** a Division of Pulmonary and Critical Care, University of North Carolina at Chapel Hill School of Medicine, Chapel Hill, NC; b Carolina Center for Pleural Diseases, University of North Carolina at Chapel Hill School of Medicine, Chapel Hill, NC; c Division of Pulmonary and Critical Care, Duke University, Durham, NC; d Division of Pulmonary, Critical Care and Sleep Medicine, University of Florida, Gainesville, FL; e Division of Pulmonary and Critical Care, Johns Hopkins University School of Medicine, Baltimore, MD; f Division of Pulmonary and Critical Care, University of Maryland School of Medicine, Baltimore, MD; g Division of Pulmonary, Critical Care and Sleep Medicine, University of New Mexico School of Medicine, Albuquerque, NM; h Division of Pulmonary, Allergy, and Critical Care Medicine, The University of Alabama at Birmingham, Birmingham, AL; i Division of Thoracic Surgery and Interventional Pulmonology, Beth Israel Deaconess Medical Center, Harvard Medical School, Boston, MA; j Division of Pulmonary and Critical Care Medicine, Weill Cornell Medicine, New York, NY; k Division of Pulmonary, Allergy, and Critical Care Medicine, Stanford University School of Medicine, Palo Alto, CA; l Division of Pulmonary, Critical Care, Hyperbaric, Allergy, and Sleep Medicine, Loma Linda University, Loma Linda, CA; m Division of Pulmonary, Critical Care, Allergy and Sleep, The University of California San Francisco, San Francisco, CA; n Division of Pulmonary, Critical Care and Sleep Medicine, Keck School of Medicine, University of Southern California, Los Angeles, CA; o Section of Interventional Pulmonology, Division of Pulmonary, Allergy, and Critical Care Medicine, Perelman School of Medicine, University of Pennsylvania, Philadelphia, PA; p Division of Pulmonary, Allergy and Critical Care Medicine, University of Pittsburgh School of Medicine, Pittsburgh, PA; q Division of Pulmonary and Critical Care Medicine, Mayo Clinic, Rochester, MN; r Division of Thoracic Surgery and Interventional Pulmonology, Swedish Cancer Institute and Center for Lung Cancer Research in Honor of Wayne Gittinger, Seattle, WA; s Division of Pulmonary and Critical Care, National Jewish Health, Denver, CO; t Division of Pulmonary and Critical Care, Yale University School of Medicine, New Haven, CT; u Division of Pulmonary and Critical Care, The University Texas MD Anderson Cancer Center, Houston, TX; v Division of Pulmonary, Critical Care, and Sleep Medicine, University of Mississippi Medical Center, Jackson, MS; w Division of Allergy, Pulmonary and Critical Care Medicine, Vanderbilt University Medical Center, Nashville, TN; x Division of Pulmonary and Critical Care, University of Utah, Salt Lake City, UT; y Interventional Pulmonology Section, Division of Pulmonary, Critical Care and Sleep Medicine, The Ohio State University Wexner Medical Center, Columbus, OH; z Division of Pulmonary and Critical Care, Virginia Commonwealth University, Richmond, VA; aa Oxford Pleural Unit, Oxford University Hospitals NHS Foundation Trust, Oxford, England; bb NIHR Oxford Biomedical Research Centre, University of Oxford, Oxford, England; cc Academic Respiratory Unit, Bristol Medical School, University of Bristol, Bristol, England

**Keywords:** bleeding, empyema, fibrinolysis, intrapleural, parapneumonic pleural effusion, IET, intrapleural fibrinolytic and enzyme therapy, tPA, tissue plasminogen activator, MIST-2, The second Multicenter Intrapleural Sepsis Trial

## Abstract

**Background:**

Combination intrapleural fibrinolytic and enzyme therapy (IET) has been established as a therapeutic option in pleural infection. Despite demonstrated efficacy, studies specifically designed and adequately powered to address complications are sparse. The safety profile, the effects of concurrent therapeutic anticoagulation, and the nature and extent of nonbleeding complications remain poorly defined.

**Research Question:**

What is the bleeding complication risk associated with IET use in pleural infection?

**Study Design and Methods:**

This was a multicenter, retrospective observational study conducted in 24 centers across the United States and the United Kingdom. Protocolized data collection for 1,851 patients treated with at least one dose of combination IET for pleural infection between January 2012 and May 2019 was undertaken. The primary outcome was the overall incidence of pleural bleeding defined using pre hoc criteria.

**Results:**

Overall, pleural bleeding occurred in 76 of 1,833 patients (4.1%; 95% CI, 3.0%-5.0%). Using a half-dose regimen (tissue plasminogen activator, 5 mg) did not change this risk significantly (6/172 [3.5%]; *P* = .68). Therapeutic anticoagulation alongside IET was associated with increased bleeding rates (19/197 [9.6%]) compared with temporarily withholding anticoagulation before administration of IET (3/118 [2.6%]; *P* = .017). As well as systemic anticoagulation, increasing RAPID score, elevated serum urea, and platelets of < 100 × 10^9^/L were associated with a significant increase in bleeding risk. However, only RAPID score and use of systemic anticoagulation were independently predictive. Apart from pain, non-bleeding complications were rare.

**Interpretation:**

IET use in pleural infection confers a low overall bleeding risk. Increased rates of pleural bleeding are associated with concurrent use of anticoagulation but can be mitigated by withholding anticoagulation before IET. Concomitant administration of IET and therapeutic anticoagulation should be avoided. Parameters related to higher IET-related bleeding have been identified that may lead to altered risk thresholds for treatment.

## Graphical Abstract

**Figure:**
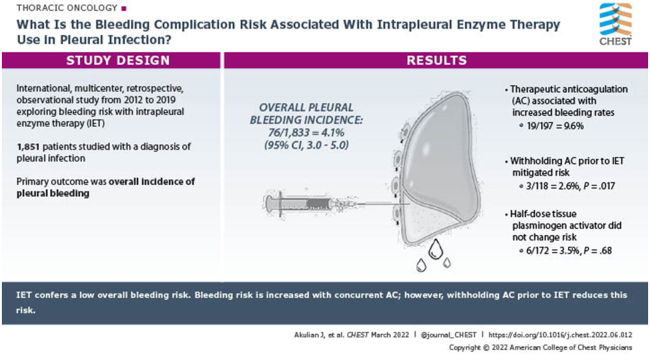

**Take-home Points**

**StudyQuestion:** What is the risk of pleural bleeding as a complication when using intrapleural fibrinolytics to manage pleural infection?

**Results:** In this largest study of intrapleural fibrinolytic and enzyme therapy (IET) in pleural infection to date, the overall incidence of bleeding complications was fewer than 1 in 20. When these occurred, most were managed without additional intervention. Apart from pain, other nonbleeding complications were rare.

**Interpretation:** Aside from specific scenarios, safety or risk of bleeding alone should no longer be a deterrent to the use of IET in pleural infection.

Pleural infection is rising in incidence^1^, ^2^, ^3^ and remains associated with prolonged hospital stays and high mortality.^4^ Combination intrapleural fibrinolytic and enzyme therapy (IET) with tissue plasminogen activator (tPA) and deoxyribonuclease (DNase) has been established as a surgery-sparing rescue treatment option.^5^ This may be required in approximately 30% to 40% of patients with pleural infection who do not respond to standard medical care with chest tube insertion and antibiotics.^4^ Given the increasing numbers of older patients, in whom frailty and comorbidities often preclude surgical options, IET has been an important addition to the therapeutic armamentarium. Despite data demonstrating efficacy,^6^ the safety of IET, in particular the potential intrapleural bleeding risk, remains a major concern for clinicians in choosing between prompt IET initiation and surgical referral.

A recent Cochrane systematic review into the use of intrapleural fibrinolytics in pleural infection concluded that data were insufficient to give a precise estimate of the overall risk of significant adverse events.^7^ The second Multicenter Intrapleural Sepsis Trial (MIST-2) recruited 52 participants in the tPA plus DNase combination arm and reported two bleeding events, giving an overall bleeding rate of 3.8%.^6^ Subsequently, a number of smaller studies reported rates of pleural bleeding with intrapleural administration of tPA (with or without DNase) in the context of pleural infection of between 1.8% and 12%.^8^, ^9^, ^10^, ^11^, ^12^, ^13^, ^14^ Other than the heterogeneity among all these studies, the key limitation is the small study populations and therefore low event rates.

Without a pre hoc definition of pleural bleeding, it is easy for a bleeding outcome to be overreported or underreported because the use of IET is known to cause hemorrhagic discoloration of pleural fluid. It remains unclear whether dose reduction alters bleeding risk, because studies evaluating such strategies have reported higher incidences of pleural bleeding (4.9%)^15^ compared with the MIST-2 study, but this was likely the result of low event rates (n = 61; three pleural bleeding events), making accurate conclusions difficult. Adequate evaluation of safety of this therapy, along with other clinically important outcomes, requires larger-scale data to achieve a significant event rate, which is difficult to achieve in the context of a single prospective pleural infection randomized controlled trial.

With these deficits in mind, this international multicenter project was designed to evaluate the indications, application, safety, and efficacy of IET for the treatment of pleural infection. The aim of this analysis specifically was to assess the overall bleeding risk and safety profile associated with IET use, including the effects of concurrent therapeutic anticoagulation and the nature and extent of nonbleeding complications. The data were also used to identify predictors of bleeding resulting from IET use.

## Study Design and Methods

### Study Design

This was a multicenter, retrospective observational study conducted in 24 centers across the United States and United Kingdom. Using Research Electronic Data Capture (Vanderbilt University), a secure web-based application for building and managing databases, a global account was developed for each center, allowing a deidentified dataset to be uploaded to the primary Research Electronic Data Capture account at the University of North Carolina.

### Ethics

Ethical and regulatory approval was obtained before recruitment began by the University of North Carolina Institutional Review Board (Identifier: 18-2906). Data were held and analyzed by University of North Carolina and the University of Oxford.

### Eligibility

The inclusion criteria were adult patients (≥ 18 years) with a diagnosis of pleural infection based on standard, internationally agreed criteria^16^ (identical to those used in large prospective, randomized controlled trials).^6^^,^^17^ These were as follows: (1) a clinical history compatible with pleural infection; (2) a pleural collection that was one of either (a) purulent, (b) gram-stain or culture positive, (c) acidic with a low pH of < 7.2, (d) low pleural fluid glucose (in the absence of an accurate pH measurement), or (e) septated pleural fluid on ultrasound (or CT scan) that is likely secondary to pleural infection; and (3) at least one dose of combination IET (both tPA and DNase) after standard medical treatment failure (as determined by the local investigator) as per local site IET protocol. In cases where the same patient received two or more courses of IET, only data for the first episode were included. Patients to whom IET was administered for recurrence of pleural infection after surgical treatment were excluded.

### Data Collection

Fourteen main data categories were included in the data collection protocol (e-Appendix 1). Specific to this analysis, patient demographics, comorbidities (including anticoagulation use), serum or pleural fluid analyses, RAPID score parameters (e-Fig 1),^18^ and details of IET therapy were assessed. The latter included dosing schedule, compliance, administration regimens, and complications. Data were captured on concurrent systemic anticoagulation use before and during IET administration.

Clinicians recorded all adverse events after IET administration. Interventions to manage pleural bleeding were captured and ranked according to a four-tier system in which each pleural bleeding event was scored according to the highest-ranking intervention required from level 1 to level 4.

### Primary and Secondary Outcomes

The primary outcome was the overall incidence of pleural bleeding. To capture only clinically significant events, the consensus definition of hemothorax was adopted in the absence of an agreed definition of pleural bleeding in the literature.^19^ The protocol mandated that for a bleeding event to be recorded, a change in pleural fluid hematocrit during therapy to ≥ 50% serum hematocrit or pleural fluid hematocrit of 25% to 50% with clinical suspicion prompting intervention was required.

Secondary outcomes included the incidence of pleural bleeding in relationship to varying dosing and administration regimens of IET, use of therapeutic systemic anticoagulation, platelets, and nonbleeding adverse events. Exploratory analysis was conducted on potential associations and predictors of bleeding events, including the RAPID score (e-Fig 1), as the only validated baseline predictor of poor clinical outcomes in pleural infection.^4^^,^^18^

### Statistical Analysis

Data are presented as mean ± SD and median (interquartile range) according to normality of data. Comparisons of proportions were conducted using the Fisher exact test (two-sided) and the χ^2^ test for variables with more than two levels. Suitable parametric and nonparametric methods were used as appropriate for other data. Data points that were missing were queried from each site and were entered as available. For missing data where centers could not provide information on data cleaning, data points were left blank and only patients with complete data were included in the final analysis. The data were analyzed using descriptive statistics, and binary outcomes were analyzed using logistic regression models. Multivariate regression models were used to identify independent predictors, with variables chosen based on the RAPID score and clinical or biological plausibility of a link to the primary outcome. Where suitable, multivariate analysis was conducted using a backward elimination approach and including parameters that were significant in univariate analysis (*P* < .05) or of clinical significance. Data analysis was carried out using SPSS version 27 software (IBM).

## Results

### Study Population

In total, 1,851 patients were enrolled in the study and 1,833 patients with complete outcome data were included in the final analysis of the primary outcome (data completion rate, 99%). Baseline characteristics of the study population were comparable with those of previously published studies of pleural infection (Table 1, e-Table 1).^4^^,^^6^ The most administered dosing regimen was that used in the MIST-2 study (10 mg tPA and 5 mg DNase, given twice daily for 3 days). Reduced dosing of tPA^15^ was used in 172 patients (9.4%), in whom the mean ± SD dose per administration was 5 ± 1 mg. Median length of treatment across the entire study population was 2 days and five doses (Table 2). Justification of dosing regimen or length of course chosen were not captured specifically, but where these were reported voluntarily, the most common reasons for stopping treatment early were pain, resolution of pleural collection, decision to proceed to surgery, or the occurrence of other complications.

**Table 1:** Baseline Characteristics of Study Population (n = 1,833)

Characteristic	Data
Age, y	57.6 ± 17.4
Male sex	1,173 (64)
Hospital-acquired infection	372 (20.3)
Small (< 15 F) chest tube	1,334 (72.8)
BMI, kg/m^2^	27.2 ± 7.35
Pleural fluid findings	
Culture positive	819 (44.7)
Pus	829 (45.3)
pH	7.12 (6.89-7.28)
Pleural fluid lactate dehydrogenase, IU	1,985 (1,246-5,374)
Radiologic loculation	1,501 (81.9)
Comorbidities	
Respiratory	472 (25.8)
Cardiac	361 (19.7)
Liver cirrhosis	89 (4.9)
Diabetes	370 (20)
End-stage kidney disease	107 (5.9)
Chemotherapy or immunosuppression	297 (16.2)
Active cancer	323 (17.7)

Data are presented as No. (%), mean ± SD, or median (interquartile range).

**Table 2:** Intrapleural Enzyme Therapy Regimen Used in the Study Population

Dosing Regimen	tPA	Deoxyribonuclease
Dose, mg	10 (10-10)	5 (5-5)
Duration, d	2 (1.75-3)	2 (1-3)
No. of doses	5 (3-6)	5 (3-6)

Administration Regimen	
Concurrent	1,388 (75.8)
Sequential	398 (21.7)
Unknown	46 (2.5)

Data are presented as No. (%) or median (interquartile range). tPA = tissue plasminogen activator.

### Incidence of Pleural Bleeding

The overall incidence of pleural bleeding in all patients treated with IET was 76 of 1,833 patients with bleeding events (4.1%; 95% CI, 3.0%-5.0%). To assess possible underlying associations of pleural bleeding, the following analyses were performed.

#### Dosing Regimen

Differences between dosing regimens were assessed in those with complete dosing details (n = 1,792). Those undergoing treatment with the MIST-2 dosing regimen showed a bleeding incidence of 66 of 1,620 patients (4.1%; 95% CI, 0.98-1.04), in comparison with a group who underwent a dose reduction strategy, in whom the bleeding incidence was 6 of 172 patients (3.5%; OR, 0.84; 95% CI, 0.37-1.9); this difference was not statistically significant (*P* = .47).

Because dose-reduction regimens may be preferred for patients with a perceived higher bleeding risk,^15^ we hypothesized that use of dose reduction was correlated with use of baseline anticoagulation. In the subgroup of patients receiving anticoagulation at baseline, 44 of 308 patients (14.3%) received dose reduction regimens vs 128 of 1,482 patients (8.6%) who were not receiving anticoagulation at baseline (*P* = .006). When the bleeding rate was compared between dosing groups corrected for use of baseline anticoagulation, no statistically significant difference was found between the full MIST-2 dose regimen and reduced dosing (OR, 1.62; 95% CI, 0.36-7.25). This analysis did not consider whether anticoagulation was withheld before administration, which is addressed separately below.

#### Administration Regimens

Concurrent (ie, tPA and DNase given together) and sequential (given separately) instillation of IET agents were compared for association with bleeding incidence. No significant difference in bleeding rate was found (concurrent administration, 55 of 1,388 patients [4.0%]; serial administration, 17 of 398 patients [4.3%]; *P* = .53).

#### Systemic Anticoagulation

Anticoagulation status during treatment was known in 1,825 of 1,833 patients (99.6%). On admission, 315 of 1,825 patients (17.3%) were receiving therapeutic anticoagulation. Use of anticoagulation was associated significantly with increased bleeding rate (no anticoagulation, 54 of 1,510 patients [3.6%]; anticoagulation, 22 of 315 patients [6.9%]; *P* = .015; OR, 1.99; 95% CI, 1.19-3.31).

Bleeding incidence was explored with respect to the effect of withholding anticoagulation before commencement of IET. Of all patients receiving systemic anticoagulation, 197 of 315 patients (63%) continued anticoagulation while being treated with IET. In patients in whom anticoagulation was withheld, median duration of withholding anticoagulation was 2 days (interquartile range, 1-4 days). A significant increase in bleeding occurrence was seen in those in whom anticoagulation was continued during IET treatment (bleeding incidence, 19 of 197 patients [9.6%]), compared with those in whom it was withheld (bleeding incidence, 3 of 118 patients [2.5%]; *P* = .008; OR, 3.76; 95% CI, 1.13-12.44).

To explore the population in whom withholding anticoagulation was deemed high risk, a further analysis of bleeding rate between the MIST-2 regimen (tPA, 10 mg) and a dose-reduction strategy (tPA, 5 mg) was conducted, correcting for withholding or continuation of anticoagulation. In those patients in whom anticoagulation was continued, the MIST-2 dosing strategy was associated with a bleeding incidence of 16 of 165 patients (9.7%) compared with 2 of 32 patients (6.3%) when using a dose-reduction strategy, but this did not reach statistical significance (*P* = .48).

#### Antiplatelet Agents

The use of therapeutic antiplatelet agents (excluding Aspirin 75 mg [UK]/Acetylsalicylic acid [ASA] 81 mg [US]) was documented in 29 patients. Of these patients, 19 continued antiplatelets during IET administration, and, in 10 patients, antiplatelets were withheld. No bleeding events occurred within this cohort; therefore, no comparative analyses were conducted.

#### Platelets

The median baseline platelet count in the study population was 275 × 10^9^/L (interquartile range, 179-397 × 10^9^/L). Logistic regression analysis was conducted to assess for the association of platelets on pleural bleeding. The predictor variable was tested a priori to verify that no violation of the assumption of the linearity of the logit had occurred. Baseline platelet count in the logistic regression analysis was found to contribute to the model (unstandardized β weight for the constant: β = –2.564; SE = 0.234; Wald = 120.517; *P* < .01; unstandardized β weight for the predictor variable: β = –0.002; SE = 0.001; Wald = 6.361; *P* = .012). The estimated OR favored a decrease of 0.2% (exp(β) = 0.99; 95% CI, 0.997-1.00) for pleural bleeding for every 1-unit increase in platelet count.

Analysis of this effect size in a clinically meaningful way was performed. It was assumed that clinician behavior would be altered significantly at platelet counts of < 50 × 10^9^/L; therefore, this small group was excluded from analysis. In patients with platelet counts of 50 to 100 × 10^9^/L, the incidence of a pleural bleeding complication (11 of 84 patients [13.1%]) was significantly greater than when platelet counts were > 100 × 10^9^/L (52 of 1,390 patients [3.7%]; *P* ≤ .001; OR, 3.50; 95% CI, 1.90-6.45). Further breakdown of this data by dosing regimen is presented in e-Table 2.

### Management of Bleeding Complications

**Figure 1:**
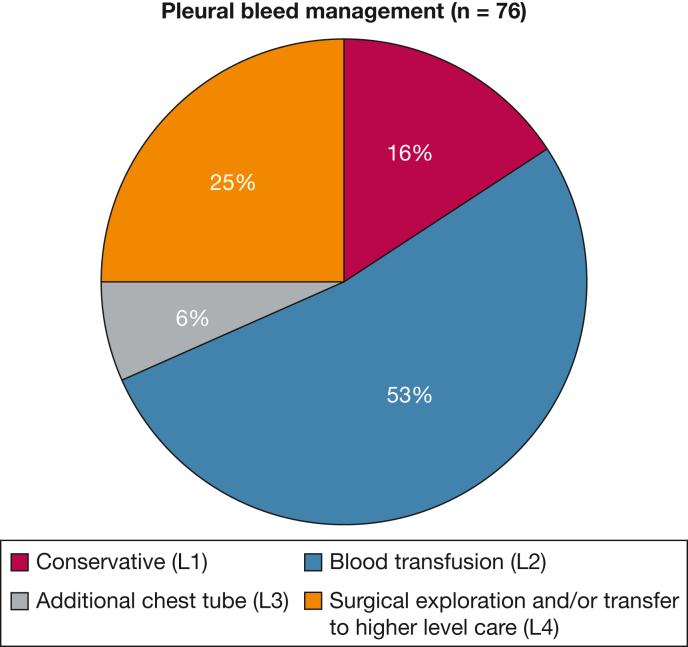
Pie chart showing management of intrapleural fibrinolytic and enzyme therapy-related pleural bleeding. L = level.

More than two-thirds of the pleural bleeding events were controlled by either withholding IET and observation alone, a blood product transfusion without the need for additional intervention, or both (Table 3, Fig 1). In 16 of 76 bleeding events (21%), the patient required surgical intervention specifically as part of bleeding management, and where details of this were available, most cases of bleeding treatment included hemothorax evacuation as well as decortication or debridement as completion treatment of the pleural infection. No documented episodes of interventional radiology-guided attempted therapies occurred.

**Table 3:** Classification of Bleeding Complications Management

Level	Management Details	No.	% of Pleural Bleeding Episodes (n = 76)	% of Study Population (n = 1,833)
1	Conservative management (stopping or temporary withholding fibrinolytics and observation)	12	15.8	0.7
2	Blood product transfusion (including correction of coagulopathy)	40	52.6	2.1
3	Additional or upsizing chest tube to manage hemothorax	5	6.6	0.3
4	Surgical exploration, transfer to higher level of care (eg, high dependency, intensive care), or both	19	25	1.0

A further analysis of whether bleeding events occurring in the context of anticoagulation use were more severe and required higher level (level 3 or 4) management was conducted. Of the 22 bleeding events that occurred in the context of anticoagulation use, 5 of 22 (22.7%) required level 3 or 4 management compared with 19 of 54 events (35%) in the nonanticoagulation group. This difference did not reach statistical significance (*P* = .36).

### Other Complications of IET Administration

Adverse events after IET administration occurred in 561 of 1,833 patients (30.6%). A breakdown of the predefined nonbleeding complications is shown in Table 4, and details of events listed as other is provided in Table 5. Pain was the most frequently reported complication (n = 224 [12.2%]). No significant difference in pain was demonstrated. No episodes of major systemic bleeding secondary to IET were reported, but death before hospital discharge was noted as an adverse event in 16 of 1,833 patients (0.9%).

**Table 4:** Main Categories of Adverse Events Reported After IET Administration

Adverse Event	No.	% of All Adverse Events (n = 561)	% of Study Population (n = 1,833)	95% CI
Pain requiring escalation of analgesics	224	39.9	12.2	11%-14%
Increased oxygen requirement	71	12.6	3.9	3%-5%
Increased level of care	44	7.8	2.4	2%-3%
Death	16	2.8	0.9	0%-1%
Hemoptysis	7	1.2	0.4	0%-1%
Other^a^	55	9.8	6.9	5%-8%

IET = intrapleural fibrinolytic and enzyme therapy.

a See Table 5.

**Table 5:** Adverse Events Reported Within Other Category

Other Adverse Events	No.	% of All Adverse Events (n = 561)	% of Study Population (n = 1,833)
Tachycardia	12	2.1	0.7
Red or bloody discoloration of fluid not meeting pleural bleeding criteria	11	1.9	0.6
Chest wall hematoma	8	1.4	0.4
Unexplained drop in hemoglobin or acute anemia without pleural bleeding	5	0.9	0.3
Air leak or bronchopleural fistula	5	0.9	0.3
Fever	4	0.7	0.2
GI bleeding	4	0.7	0.2
Hypotension	3	0.5	0.2
Allergic or hypersensitivity reaction	3	0.5	0.2

### RAPID Score as a Predictor of IET-Related Pleural Bleeding

Complete RAPID score data were available for 1,494 of 1,833 patients (81.5%). Distribution of the RAPID score was comparable with the external validation cohort (Pleural Infection Longitudinal Outcome Study; PILOT) (e-Figs 2, 3). The association between RAPID categorization and bleeding risk was explored using a multinomial logistic regression analysis using three RAPID risk categories as in the previous publications (low [RAPID score, 0-2], medium [RAPID score, 3-4], and high [RAPID score, 5-7]). Bleeding frequency was associated significantly with baseline RAPID risk category (χ^2^ 2 *df* = 15.4; *P* < .0001) (Table 6).

**Table 6:** Bleeding Events by RAPID Score (Low Category as the Reference Group)

RAPID Score Category	No.	No. of Bleeding Events	Proportion of Bleeding Events (%)	R (95% CI)
Low (0-2)	447	12	2.6	Not applicable
Medium (3-4)	692	25	3.5	1.35 (0.67-2.71)
High (5-7)	355	31	8	3.25 (1.65-6.43)

Within components of the RAPID score, it was hypothesized that age, urea level, and albumin level were the most likely contributors to bleeding risk, based on biological plausibility. Analysis was conducted to assess the independent predictive ability of these variables using multiple logistic regression. The overall three-variable model significantly predicted bleeding (*F* (3, 1,499) = 3.13; *P* = .025), but urea level was the only significant independent predictor (urea: β = 0.068; *P* = .009; age: β = 0.027; *P* = .299; albumin: β = –0.011; *P* = .663).

### Other Predictors of Pleural Bleeding

A univariate regression analysis was performed for all factors where an association with pleural bleeding was biologically plausible, including the individual components of the RAPID score (e-Table 3). Receiving therapeutic anticoagulation on admission, serum urea level, platelet level, and final RAPID score all predicted a greater likelihood of pleural bleeding. A multivariate logistic regression model was then performed (beginning with all univariate factors), and backward elimination (*P* < .1) was used to identify independent predictors of a pleural bleeding outcome. RAPID score category and use of active anticoagulation were the only independent predictors of a pleural bleeding outcome (Table 7). The full model is shown in e-Table 4.

**Table 7:** Independent Predictors of Pleural Bleeding Outcome (Final Model of the Multivariate Regression Using Backward Elimination)

Variable	*P* Value	OR	95% CI
Active anticoagulation	.048	1.80	1.01-3.23
RAPID category	.005	1.72	1.17-2.51

Further analyses were undertaken to explore bleeding incidence in patients with liver cirrhosis and end-stage renal disease, correcting for dosing regimen used. Liver cirrhosis was not associated with a significant increase in bleeding regardless of dosing regimen (tPA, 5 mg: *P* = .49; tPA, 10 mg: *P* = .20). In the context of patients treated with full-dose tPA, end-stage renal disease was associated with an increased incidence of pleural bleeding (7 of 79 patients [8.9%] vs 51 of 1,301 patients [3.9%]; *P* = .037; OR, 2.4; 95% CI, 1.04-5.43). However, in the population treated with a dose-reduction strategy (tPA, 5 mg), end-stage renal disease was not associated with a statistically significant increase in pleural bleeding events (0 of 15 patients [0%] vs 6 of 142 patients [4.2%]; *P* = .42; OR, 0.96; 95% CI, 0.92-0.99).

## Discussion

To our knowledge, this is the largest study to date of combination IET in pleural infection, and the only study to use pre hoc criteria to define pleural bleeding events. The bleeding risk of 4.1% found in this data is comparable with the original bleeding incidence of 3.8% reported in the MIST-2 study. The IET dosing regimen in MIST-2 was chosen empirically but nonetheless to date remains the only dosing regimen to have been tested in a randomized placebo-controlled trial. These data demonstrated that reducing the dose of tPA in routine use (all comers) is not associated with a decrease in pleural bleeding incidence, perhaps suggesting that the dose effect for bleeding is different in intrapleural vs IV use.^20^^,^^21^

Systemic anticoagulation is associated with increased intrapleural bleeding, but temporarily omitting treatment before IET (or an international normalized ratio of < 2 in the context of warfarin) seems to mitigate this risk. Our data suggest that concomitant administration of systemic anticoagulants and intrapleural fibrinolytics requires careful consideration, because this increased the risk of intrapleural bleeding by fourfold. In some clinical scenarios, risks of withholding anticoagulation may be unacceptably high (eg, metallic heart valves or recent venous thromboembolic events). In such cases, a cautious approach should be adopted in the use of IET, such as more easily reversible anticoagulation, for example, heparin as opposed to direct oral anticoagulant, or consideration of alternative interventions to IET, such as surgical approaches or intrapleural saline irrigation,^22^^,^^23^ accepting the inferior evidence base for the latter.

In patients in whom pleural bleeding occurred with IET, most events resolved with observation or with blood product transfusion. Nonetheless, almost one-third of bleeding events did require pleural or surgical intervention, and this information should inform clinical decision-making and patient discussions in consenting to therapy. Of other complications, pain was the most frequent nonbleeding event; hence, consent for this likely side effect and premedication with appropriate analgesia should be considered to improve tolerability and compliance.

The analysis of predictors of pleural bleeding has shown that the RAPID score, as an independent predictor, allows clinicians to make a direct estimation of bleeding risk from IET at presentation and is not subject to the variability of serum urea and platelet levels. This is a novel use of the RAPID score derived from this study.

Our data provide reassurance that age does not seem to confer an increased bleeding risk. This is particularly relevant because IET use is targeted with increasing frequency in older, frailer patients, who are deemed to be high-risk surgical candidates and in whom IET may represent the only viable rescue treatment option.

This study has several strengths. It is more than 10 times larger than the previous largest study evaluating the use of IET in the treatment of pleural infection. At this study size, we can provide precise estimates on frequency of events as reflected in the narrow 95% CIs, and these data can now be used to provide precise information to clinicians for decision-making and the consent process. Our study included very high rates of data completeness and identical dataset collection across multiple centers capturing global practices and therefore has strong external validity. The study population represents all-comers as opposed to the carefully selected patients enrolled into interventional clinical trials. This is the first study to our knowledge to use an a priori objective definition of the key clinical event (pleural bleeding), adding to the robustness of reporting for the primary outcome. This aspect is important for future studies, because IET use is recognized to be associated with red or blood discoloration of drained pleural fluid as lysis of fibrin strands occurs, and IET use results in increased pleural fluid formation and drainage.^24^ These factors in combination can cause alarm and may be assigned mistakenly to pleural bleeding.

This study has limitations. It was a retrospective study, and although the large study size and event rate mitigate against this to some extent, this is not equivalent to randomized controlled trial data with consecutive patient recruitment and therefore is subject to some selection bias. This can be seen, for example, in the nonbleeding adverse events in which it is likely that some events, such as death, although exceedingly rare, may have been caused by factors other than IET administration. Similarly, other adverse events (such as red discoloration of fluid) are likely to have been underreported because most clinicians would not consider this to be a true adverse event. In the absence of non-IET-treated control participants, it is challenging to assign causation to some findings. Prophylactic anticoagulation was not addressed in this study; however, previous studies have not shown an association with increased bleeding risk.^25^ The low number of patients receiving antiplatelet agents precluded the ability to study this subgroup in detail, and therefore, until further data are available, the authors suggest that antiplatelet agents (other than aspirin 75 mg/ASA 81 mg) are withheld (if clinically appropriate) before IET administration.

## Interpretation

This is the largest study to date, to our knowledge, of IET use in pleural infection, confirming a low bleeding risk. Although bleeding risk is increased with concurrent anticoagulation, withholding anticoagulation before IET therapy reduces this risk, and caution is advised for concomitant administration of IET and therapeutic-dose systemic anticoagulation. An increased bleeding risk is associated with increasing RAPID score, elevated serum urea level, and serum platelet count of < 100 × 10^9^/L. The RAPID score and the use of active systemic anticoagulation are independent predictors of IET-related bleeding risk in pleural infection.

## Supplementary Data

e-Online Data

e-Online Data
