# Supplementary material for: Bleeding Risk With Combination Intrapleural Fibrinolytic and Enzyme Therapy in Pleural Infection: An International, Multicenter, Retrospective Cohort Study
Source: Chest. 2022 Jun 16;162(6):1384–92. doi: 10.1016/j.chest.2022.06.008 (PMC9773231; doi:10.1016/j.chest.2022.06.008)

**RAPID score parameters (e-Figure 1)**


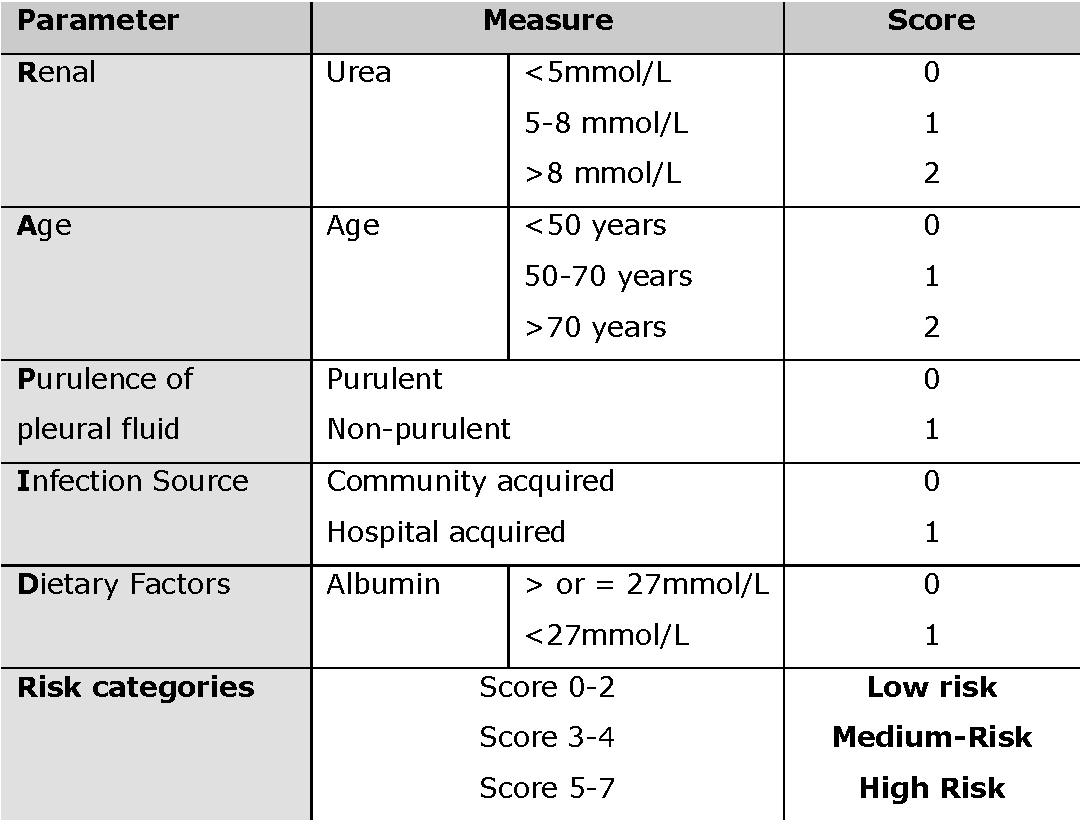


**Distribution of the RAPID score in the PILOT study cohort (e-Figure 2)**


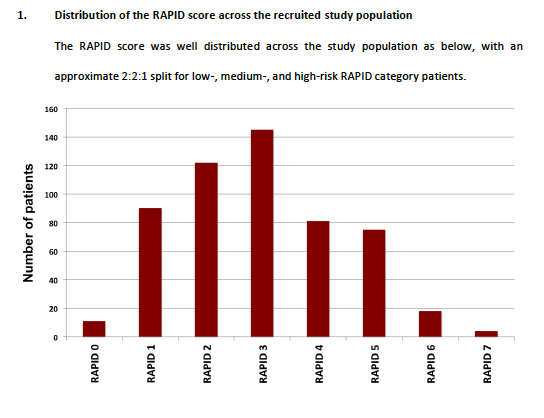


**Distribution of the RAPID score in the IPOG study cohort (e-Figure 3)**


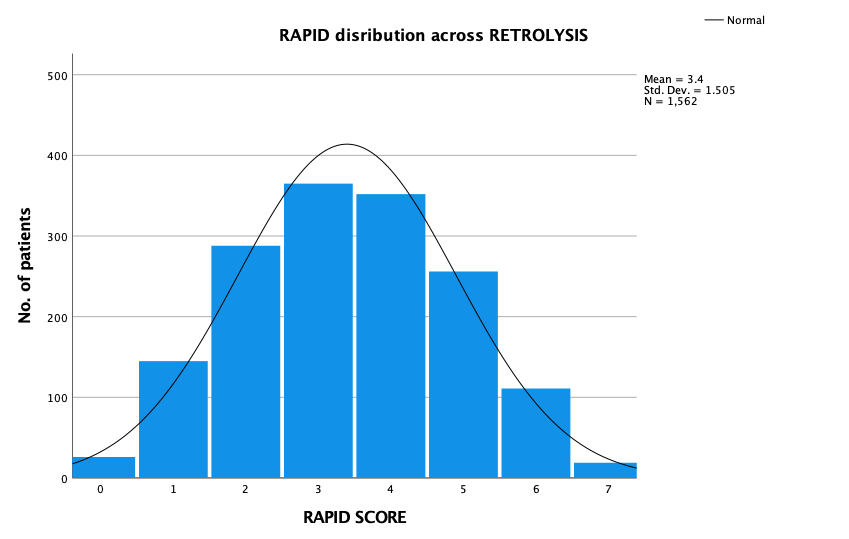

Supplement: e-Online Data [file mmc1.docx]
