# Supplementary material for: Bleeding Risk With Combination Intrapleural Fibrinolytic and Enzyme Therapy in Pleural Infection: An International, Multicenter, Retrospective Cohort Study
Source: Chest. 2022 Jun 16;162(6):1384–92. doi: 10.1016/j.chest.2022.06.008 (PMC9773231; doi:10.1016/j.chest.2022.06.008)
Supplement: e-Online Data [file mmc2.docx]

**Bleeding risk with combination intrapleural fibrinolytic and enzyme therapy in pleural infection – an international, multicenter, retrospective cohort study**

**Online Supplement**

1. **Data points collected by the Interventional Pulmonology Outcomes Group (IPOG)**

Patient demographics

1. Age

2. Sex

3. Race

4. Date of hospital admission

5. Date of chest tube placement

6. Date of chest tube removal

7. Date of hospital discharge

8. Co-morbidities

a. Lung Disease

i. COPD

ii. ILD

iii. Cystic fibrosis

iv. Lung Cancer

v. Other

b. Non-lung co-morbidities

i. DM

ii. Malnutrition

iii. s/p Transplantation

iv. Active Malignancy

v. Use of Immunosuppressive Medication

vi. Chemotherapy

c. Anticoagulation

i. Therapeutic Heparin

ii. DoAC

iii. Coumadin/warfarin

d. Antiplatelets

iv. Asa 325 mg

v. Clopidogrel

vi. Other (do not include Aspirin 75/Asa 81 or prophylactic LMWH)

9. Radiographic Features (CT): Review by either radiologist or data abstracter acceptable.

a. Pleural Thickening (> 2 mm)

b. Loculation

c. Internal Septation

d. Abscess or necrotizing pneumonia

10. Serum (Peak value for all but platelets. Nadir for platelets. Within 7 days of lytic administration)

a. Bun

b. PT/INR

c. PTT

d. WBC

e. Platelets (lowest)

11. Pleural Fluid Analysis

a. Diagnosis (empyema, CPPE etc…)

b. Culture (+/- and results)

c. Gram stain (+/- and results)

d. Total Protein (g/dL)

e. LDH (U/L)

f. pH

g. Glucose (mg/dL)

h. Fluid description

12. Chest Tube and tPA/Dornase administration

a. Date of Initial Chest Tube Placement

b. Date of Initial Chest Tube Removal

c. Initial Chest Tube Size (F)

d. Doses TPA/DNAse

e. Frequency of administration

f. Total number of doses

g. Timing of lytics (concurrent/serial)

h. Total chest tube output

i. Pre Lytics

ii. After Initiation of Lytics

i. Complications

i. Pain (Requiring intervention)

ii. Increased level of care

iii. Increase O2 Requirement

iv. Death

v. Hemoptysis

vi. Initial Tube Dislodgement

vii. Hemorrhage (Pleural fluid Hct ≥ 50% of serum Hct, or 25-50% of serum Hct with clinical suspicion)

viii. If Hemorrhage How Managed

ix. Other

13. Outcomes

a. Date of Hospital Admission

b. Date of Hospital Discharge

c. Additional chest tube placement

i. #

ii. Size(s)

iii. Reason for additional tubes (Ongoing Sepsis, Significant Undrained Focus of Fluid)

d. Additional doses of lytics (tPA+Dornase)

i. #

ii. Reason for additional doses of lytics (Ongoing Sepsis, Significant Undrained Focus of Fluid)

e. Surgical referral

i. Performed

1. Open

2. Minimally invasive thoracic surgery (MITS)

14. Mortality

a. 30-day

b. 90-day

c. Alive > 90 Days

1. **Comparison of demographics and baseline characteristics of the study population with that of the MIST-2 randomised controlled trial (Rahman et al NEJM 2011) and the Pleural Infection Longitudinal Outcomes Study (PILOT) (Corcoran et al ERJ 2020). The variables highlighted yellow indicate components of the RAPID score.**

e-Table 1

|  | MIST-2 (n=210) | PILOT (n=547) | IPOG (n=1833) |
| --- | --- | --- | --- |
| Mean age (SD) | 58.8 (18.1) | 60 (SD 18) | 57.6 (17.4) |
| Male | 151 (71.9) | 385 (71%) | 1173 (64%) |
| Hospital Acquired Infection - n (%) | 28 (13.3%) | 259 (48%) | 372 (20.3%) |
|  |  |  |  |
| Small (<15F) chest tube | NR | 309 (70%) | 1334 (72.8%) |
|  |  |  |  |
| Mean pH | 6.9 (SD 0.3) | 7.00 (0.5) | 7.12 (SD 0.45) |
| Pus n (%) | 102 (48.6%) | 222 (41%) | 829 (45.3%) |
| Median PF LDH (IQR) | NR | 1968 (4063) | 1984.5 (4128) |
|  |  |  |  |
| Mean WCC (SD) | NR | 18.2 (20.8) | 17.87 (11.7) |
| Median urea (IQR) | 5.0 (4.2) | 4.8 (3.95) | 6.4 (7.14) |
| Mean albumin (SD) | 31.5 (7.8) | 28.5 (7.5) | 27.5 (15.6) |
| Median creatinine (IQR) | 78 (66-97) | 67 (30) |  |
|  |  |  |  |
| Comorbidities |  |  |  |
| Respiratory problems | 51 (28.3%) | 150 (27.6%) | 472 (25.8%) |
| Cardiac problems | 56 (30.6%) | 84 (15.4%) | 361 (19.7%) |
| Cirrhosis | 23 (12.7%) | 28 (5%) | 89 (4.9%) |
| Diabetes | 29 (16%) | 77 (14%) | 370 (20%) |

1. **Difference in bleed complications by platelet count and IET dosing regimen**

**e-Table 2**

| IET Dosing regimen | Platelet count | n | Bleed events n (%) | p-value | OR (95% CI) |
| --- | --- | --- | --- | --- | --- |
| Half dose | 50-100 | 7 | 1 (14.3) | 0.16 | 4.03 (0.54 - 30.0) |
|  | >100 | 141 | 5 (3.5) |  |  |
| Full dose | 50-100 | 77 | 10 (13.0) | <0.001 | 3.45 (1.81 - 6.56) |
|  | >100 | 1249 | 47 (3.8) |  |  |

1. **Pleural bleed outcome prediction**

**e-Table 3 – Univariate regression analysis of pleural bleed outcome predictors**

| Variable | df | p-value | Odds ratio |
| --- | --- | --- | --- |
|  |  |  |  |
| Patient age | 1 | 0.13 | 1.01 |
| BMI (kg/m2) | 1 | 0.61 | 0.99 |
| Liver cirrhosis | 1 | 0.17 | 0.18 |
| End stage renal disease | 1 | 0.14 | 1.83 |
| Active malignancy | 1 | 0.66 | 0.86 |
| Active chemotherapy | 1 | 0.19 | 0.45 |
| Active anticoagulation*$ | 1 | 0.01 | 1.99 |
| RAPID category*$ | 3 | 0.03 | - |
| Serum urea** | 1 | 0.003 | 1.01 |
| Serum albumin (g/dL) | 1 | 0.38 | 0.85 |
| Serum platelets* | 1 | 0.035 | 0.99 |
| Hospital acquired infection | 1 | 0.12 | 1.54 |
| Absence of pus | 1 | 0.70 | 0.90 |

*p<0.05 **p<0.01

$ Statistically significant in the multivariate model

**e-Table 4 – Multivariate regression analysis for pleural bleeding following IET using backward elimination (standardized regression coefficients)**

| **Variables** | **Model** | | | | | | | | | |
| --- | --- | --- | --- | --- | --- | --- | --- | --- | --- | --- |
|  | **1** | **2** | **3** | **4** | **5** | **6** | **7** | **8** | **9** | **10** |
| Patient age | 0.004 | 0.004 | - | - | - | - | - | - | - | - |
| BMI (kg/m^2^) | -0.008 | -0.008 | -0.008 | -0.008 | -0.008 | - | - | - | - | - |
| Liver cirrhosis | -1.646 | -1.646 | -1.642 | -1.631 | -1.611 | -1.602 | -1.597 | -1.573 | - | - |
| End stage Renal Disease | 0.243 | 0.244 | 0.230 | 0.250 | - | - | - | - | - | - |
| Active malignancy | -0.194 | -0.194 | -0.171 | - | - | - | - | - | - | - |
| Active chemotherapy | -0.747 | -0.746 | -0.740 | -0.863 | -0.871 | -0.865 | -0.895 | - | - | - |
| Active anticoagulation | 0.497 | 0.497 | 0.510 | 0.511 | 0.508 | 0.507 | 0.516 | 0.503* | 0.545 | **0.589*** |
| RAPID category | 0.388 | 0.394 | 0.448* | 0.443* | 0.436* | 0.442* | 0.493* | 0.502 | 0.485* | **0.540**** |
| Serum urea | 0.004 | 0.004 | 0.004 | 0.004 | 0.004 | 0.004 | - | - | - | - |
| Serum albumin (g/dL) | -0.012 | - | - | - | - | - | - | - | - | - |
| Serum platelets | -0.002 | -0.002 | -0.002 | -0.002 | -0.002 | -0.002 | -0.002* | -0.002 | -0.002 | - |
| Hospital acquired infection | 0.363 | 0.356 | 0.365 | 0.370 | 0.335 | 0.322 | 0.318 | - |  |  |
| Absence of pus | -0.066 | -0.072 | - | - | - | - | - | - | - | - |

*p $\leq$ 0.05 **p $\leq$ 0.01
